# ZjSEP3 modulates flowering time by regulating the *LHY* promoter

**DOI:** 10.1186/s12870-021-03305-x

**Published:** 2021-11-11

**Authors:** Weilin Gao, Liman Zhang, Jiurui Wang, Zhiguo Liu, Yao Zhang, Chaoling Xue, Mengjun Liu, Jin Zhao

**Affiliations:** 1 College of Life Science, Hebei Agricultural University, Baoding, 071000 China; 2 College of Forestry, Hebei Agricultural University, Baoding, 071000 China; 3 Research Center of Chinese Jujube, College of Horticulture, Hebei Agricultural University, Baoding, 071000 China

**Keywords:** *Ziziphus jujuba* Mill., *ZjSEP3*, Early flowering, Transcriptional factor, *LHY* promoter, CArG-box

## Abstract

**Background:**

*SEPALLATA3* (*SEP3*), which is conserved across various plant species, plays essential and various roles in flower and fruit development. However, the regulatory network of the role of SEP3 in flowering time at the molecular level remained unclear.

**Results:**

Here, we investigated that *SEP3* in *Ziziphus jujuba* Mill. (*ZjSEP3*) was expressed in four floral organs and exhibited strong transcriptional activation activity. *ZjSEP3* transgenic Arabidopsis showed an early-flowering phenotype and altered the expression of some genes related to flowering. Among them, the expression of LATE ELONGATED HYPOCOTYL (*AtLHY*), the key gene of circadian rhythms, was significantly suppressed. Yeast one-hybrid (Y1H) and electrophoretic mobility shift assays (EMSAs) further verified that ZjSEP3 inhibited the transcription of *AtLHY* by binding to the CArG-boxes in its promoter. Moreover, ZjSEP3 also could bind to the *ZjLHY* promoter and the conserved binding regions of ZjSEP3 were found in the *LHY* promoter of various plant species. The ectopic regulatory pathway of ZjSEP3-AtLHY was further supported by the ability of 35S::*AtLHY* to rescue the early-flowering phenotype in *ZjSEP3* transgenic plants. In *ZjSEP3* transgenic plants, total chlorophyll content and the expression of genes involved in chlorophyll synthesis increased during vegetative stages, which should contribute to its early flowering and relate to the regulatory of *AtLHY*.

**Conclusion:**

Overall, ZjSEP3-*AtLHY* pathway represents a novel regulatory mechanism that is involved in the regulation of flowering time.

**Supplementary Information:**

The online version contains supplementary material available at 10.1186/s12870-021-03305-x.

## Background

Floral organ identities have been explained by the classic ABC model in the model plant Arabidopsis [1, 2]. Researchers have subsequently discovered that floral organs are regulated by E-class genes [3]. E-class (*SEPALLATA*, *SEP*) genes play essential and various roles in reproductive organ development [4–6]. The effects of SEP-class genes on the regulation of flowering time vary. In peach (*Prunus persica*), only two of five MADS-box genes, *PrpMADS5* and *PrpMADS7*, can induce early blossoming [7]. In Arabidopsis, E-class genes comprise four members, *SEP1* (*AGL2*), *SEP2* (*AGL4*), *SEP3* (*AGL9*) and *SEP4* (*AGL3*) [8]. Overexpression of *SEP3* in Arabidopsis can stimulate early flowering [9], and 35S::*LMADS3* (SEP3-like gene of lily) transgenic Arabidopsis plants also exhibit an early-flowering phenotype [10]. *SEP3* genes of some other species have also been found to regulate flowering time [11–14]. However, little is known about the regulatory network of the role of *SEP3* in flowering time at the molecular level.

As a transcription factor, SEP3 is a multifunctional protein involving many developmental processes [15–18]. SEP3 could modulate auxin signaling pathway by targeting some related genes, such as *ARF*, *PIN4* and *PID* [18]. SEP3 and APETALA1 (AP1) were shown to directly interact with SEUSS (SEU), which play crucial roles in modulating the function of the class C gene [19, 20]. And SEP3 modulate the expression of other floral homeotic genes by binding to their promoters, and it can regulate *AP3* expression by binding to CArG boxes in its promoter [18, 21].

Chinese jujube (*Ziziphus jujuba* Mill.) is a member of the Rhamnaceae and a major dry fruit crop species and has been cultivated in China for more than 3000 years. Jujube fruits have high nutritional value, including vitamin C and amino acids. Moreover, jujube fruits and seeds are traditional herbal medicines in Asia. Compared with other perennial fruit tree species, jujube has a short juvenile phase and can blossom the same year it is planted. Floral bud differentiation occurs many times during the same year of flowering, and this characteristic is very unique among perennial fruit trees. Jujube genome sequencing has provided valuable data resources for elucidating the molecular mechanisms governing the biological characteristics of this species [22, 23].

In our previous study, we identified 52 MADS-box genes and found three SEP-class genes in the jujube genome [24]. Among them, *ZjSEP3* was found to be expressed in all four whorls of the flower organs, and 35S::*ZjSEP3* transgenic Arabidopsis exhibited an early-flowering phenotype. The floral regulators *GIGANTEA* (*GI*), *CONSTANS* (*CO*), and *FLOWERING LOCUS T* (*FT*) play key roles in the photoperiodic flowering responses [25–28], the pathways of which are highly conserved in plants. *LEAFY* (*LFY*), together with *FT*, can promote flowering. Thus, the expression of above genes related to flowering were examined in 35S::ZjSEP3 transgenic Arabidopsis. It found that ZjSEP3 could alter the expression of these genes and inhibited the transcription of *LATE ELONGATED HYPOCOTYL* (*LHY*) by binding to its promoter region. LHY, a MYB protein, plays crucial roles in the maintenance of the circadian rhythm [29–32]. The transcriptional regulation of the *LHY* gene was the key to the structure of the circadian oscillator, integrating information from multiple regulatory pathways [25, 33–35]. Here, we demonstrated a novel interaction network in which SEP3 is a positive regulator of flowering time by regulating *LHY*.

## Results

### Phylogenetic tree construction and conserved motifs of *ZjSEP3*

**Fig. 1:**
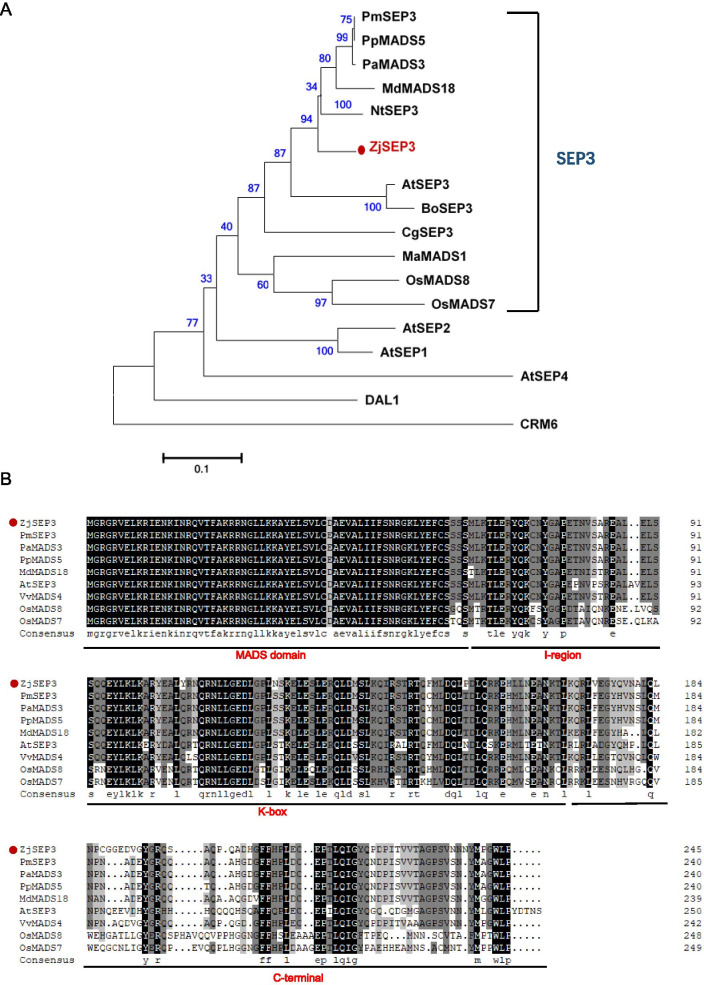
Phylogenetic tree and sequence alignment of ZjSEP3 and other homologous proteins. **A** Phylogenetic tree of ZjSEP3 and homologous proteins from other species. Note: The red circle indicates ZjSEP3**.** Arabidopsis AtSEP1, 2, and 4; DAL1; and CRM6 were used as outgroups. The scale bar represents 0.1 substitutions per site. PmSEP3 (*Prunus mume*), XP_008222191.1; PpMADS5 (*Prunus persica*), ABO27621.1; PaMADS3 (*Prunus avium*), AEN75254.1; MdMADS18 (*Malus domestica*), ADL36740.1; NtSEP3 (*Nicotiana tomentosiformis*), XP_009600809.1; AtSEP3 (*Arabidopsis thaliana*), NP_564214.2; BoSEP3 (*Brassica oleracea var. oleracea*), XP_013638953.1; CgSEP3 (*Cymbidium goeringii*), APY18453.1; MaMADS1 (*Musa acuminata* subsp. malaccensis), XP_009384296.1; OsMADS8 (*Oryza sativa*), Q9SAR1.1; OsMADS7 (*O. sativa*), Q0J466.2; AtSEP1 (*A. thaliana*), NP_001119230.1; AtSEP2 (*A. thaliana*), NP_186880.1; AtSEP4 (*A. thaliana*), NP_178466.1; DAL1 (*Picea abies*), X80902; CRM6 (*Ceratopteris pteridoides*), Y08242. **B** Multiple sequence alignment of ZjSEP3 and eight homologous proteins from other species. Note: The horizontal lines indicate the four conserved domains. VvMADS4 (*Vitis vinifera*), AAM21344.1; OsMADS8 (*Oryza sativa*), Q9SAR1.1; OsMADS7 (*O. sativa*), Q0J466.2

To better understand the relation of ZjSEP3 and SEP proteins from other plant species, a phylogenetic tree was constructed in this study (Fig. 1A), showing that ZjSEP3 clustered with the SEP3 proteins of other species. Multiple sequence alignment of ZjSEP3 and its homologs from eight other species also revealed that these proteins contained highly conserved MADS and K domains (Fig. 1B), indicating that SEP3 is conserved in the evolution of plants. Moreover, the above comparisons also revealed that ZjSEP3 shared high identity with that of Rosacea species, e.g., 88.98% identity with PmSEP3 (*Prunus mume*, XP_008222191.1) and 88.57% identity with PpMADS5 (*P. persica*, ABO27621.1).

### Expression patterns of *ZjSEP3* during jujube flower development

**Fig. 2:**
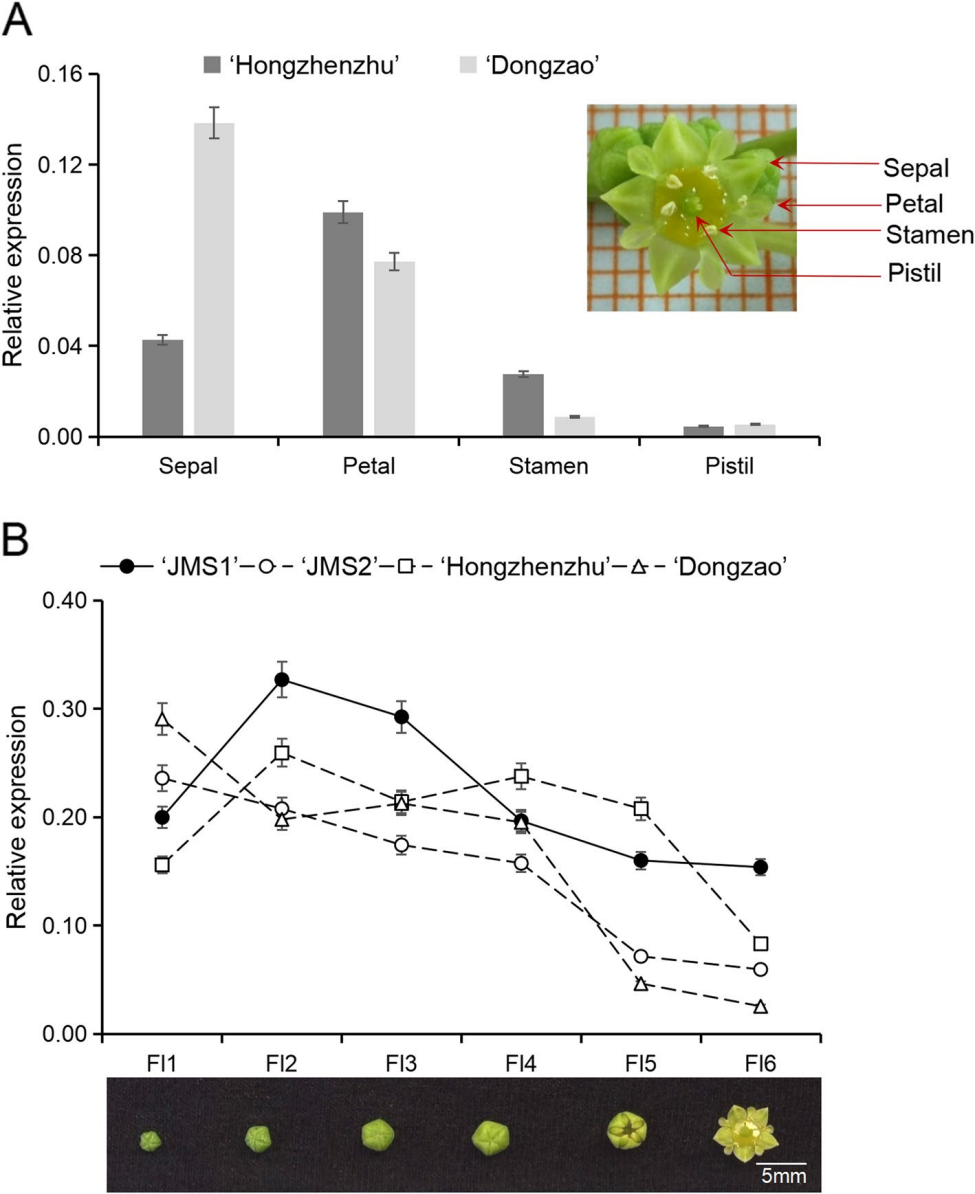
Expression patterns of *ZjSEP3* in the flowers of *Ziziphus jujuba*. **A** Expression patterns of *ZjSEP3* in different floral organs of *Z. jujuba*. **B** Expression patterns of *ZjSEP3* during jujube flower development. Fl1-Fl6: very small buds (Fl1), small buds (Fl2), medium buds (Fl3), large buds (Fl4), split buds (Fl5), open flowers (Fl6)

Our previous study showed that *ZjSEP3* was expressed mainly in floral tissues compared with different vegetative and reproductive tissues [24]. To further investigate the expression patterns of *ZjSEP3*, its expression in different floral organs and during different development stages of flowers was analyzed in jujube varieties. In ‘Hongzhenzhu’ the expressions of *ZjSEP3* from the highest to the lowest were petal, sepal, stamen and pistil, while in ‘Dongzao’ its expressions from the highest to the lowest were sepal, petal, stamen and pistil (Fig. 2A). Overall, *ZjSEP3* was expressed in four floral organs and predominantly expressed in the sepals and petals. During flower development, *ZjSEP3* expression was increased at the FI2 stage in ‘JMS1’ and ‘Hongzhenzhu’ and decreased in ‘JMS2’ and ‘Dongzao’. Then its expression dropped continuously in different varieties except ‘Hongzhenzhu’. In ‘Hongzhenzhu’, *ZjSEP3* expression was unregulated slightly from FI2 to FI4 stages. At the FI6 stage, its expression in ‘JMS1’ was higher compared to the others. On the whole, its expression was highly in the early development stages of jujube flowers and dropped considerably in the last two stages in four varieties (Fig. 2B), suggesting that it plays some functions during the early development of jujube flower.

### Subcellular localization of ZjSEP3 and its transcriptional function

**Fig. 3:**
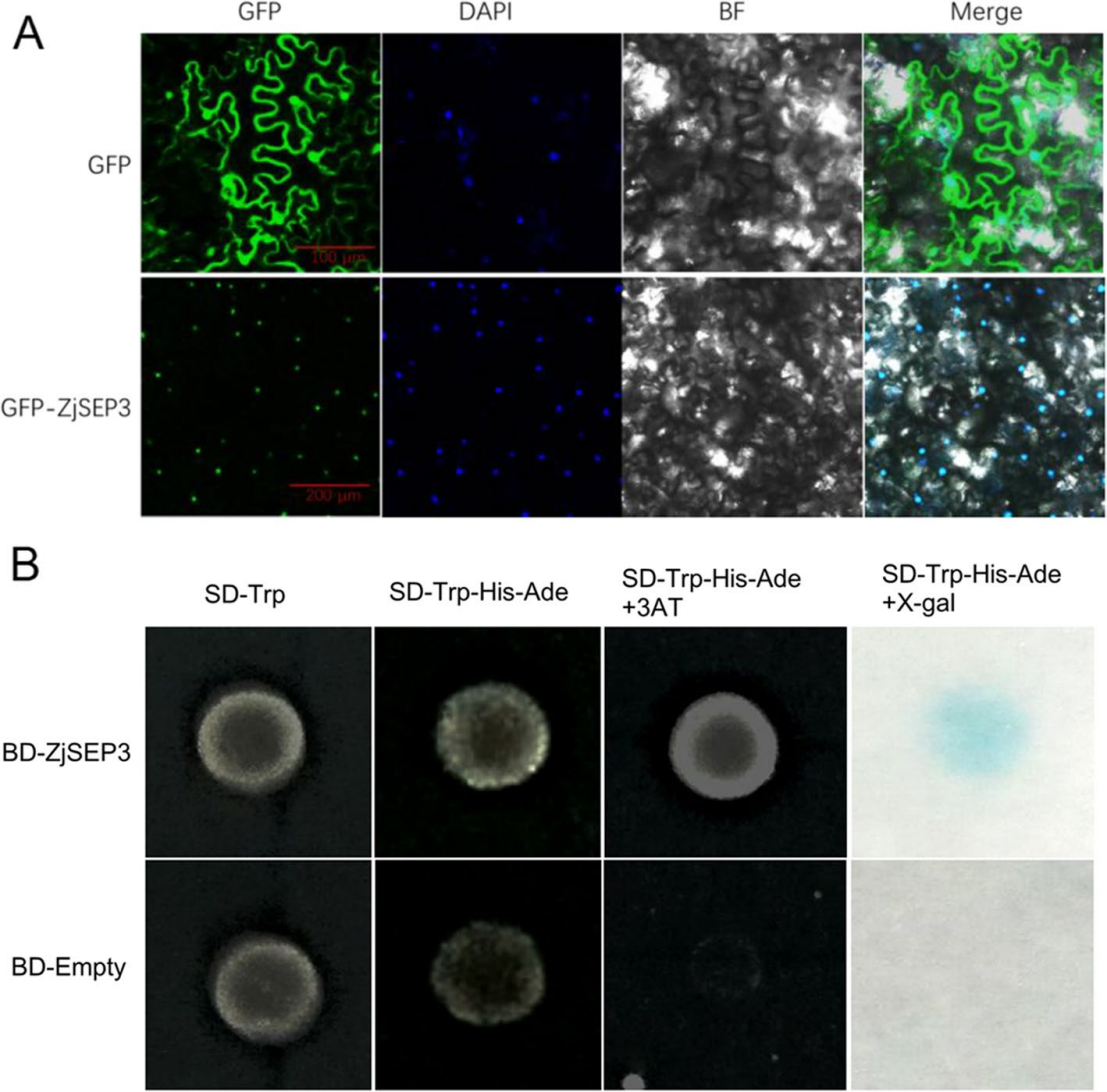
Subcellular localization of ZjSEP3 and its transcriptional function. **A** Subcellular localization of ZjSEP3 in agro-infiltrated *Nicotiana benthamiana* leaves. The vector was 35S-ZjSEP3-GFP carrying Agrobacterium GV3101. **B** Transcriptional function of ZjSEP3. BD-empty constructs served as negative controls

ZjSEP3 was predicted to localize to the nucleus, and confocal green fluorescence imaging revealed that ZjSEP3-GFP fusion protein was truly located in the nucleus, whereas free GFPs and 35S::GFP constructs were distributed throughout the whole cell (Fig. 3A). At the same time, the transcriptional activity of ZjSEP3 in vivo was further confirmed by yeast one-hybrid assays (Fig. 3B).

### ZjSEP3 affects flower development

**Fig. 4:**
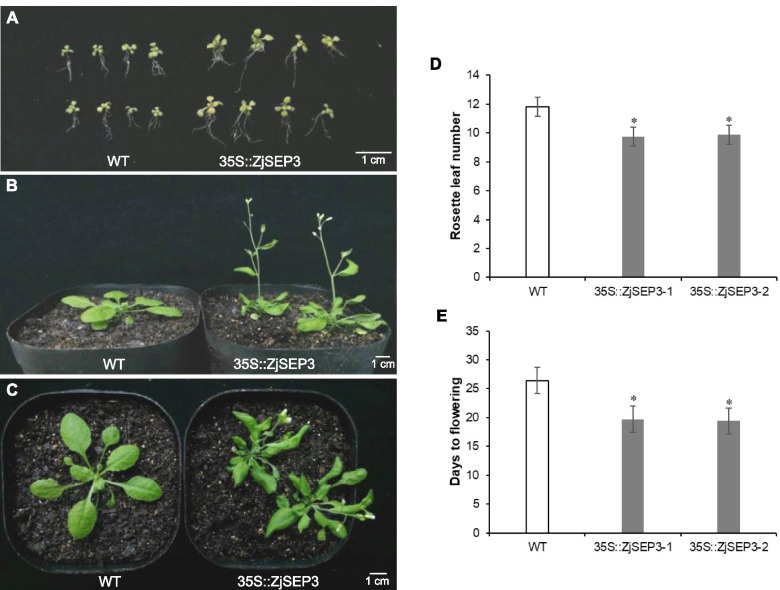
Phenotypic analyses of 35S::*ZjSEP3* transgenic plants. **A** 35S::*ZjSEP3* transgenic plants were larger than wild-type (WT) plants at the seedling stage. **B** Compared with WT plants, 35S::*ZjSEP3* transgenic plants flowered earlier. **C** The rosette leaves of 35S::*ZjSEP3* transgenic plants are curled. **D**, **E** Rosette leaf number and days of WT and 35S::*ZjSEP3* transgenic plants until flowering. * represents significantly different from the WT plants at *P* < 0.05 level. 35S::*ZjSEP3–1* and 35S::*ZjSEP3–2* represent two transgenic lines

To further elucidate the function of *ZjSEP3*, we ectopically overexpressed this gene in Arabidopsis. Phenotypic characterization revealed that, compared with the controls, the 35S::*ZjSEP3* lines displayed macroscopic differences (Fig. 4). The overexpression of *ZjSEP3* significantly affected the development of Arabidopsis plants, which plants were bigger at the seedling stage than wild-type (WT) plants (Fig. 4A, B, Fig. S1); in addition, the leaves of the 35S::*ZjSEP3* plants became curled (Fig. 4C), which was consistent with previously studies for 35S::*SEP3* plants [15, 36]. The statistical results showed that, compared with that of WT plants, the flowering time of the 35S::*ZjSEP3* plants was clearly accelerated, and which also had fewer rosette leaves when flowering (Fig. 4D, E).

### ZjSEP3 affects the expression of genes related to flower development

**Fig. 5:**
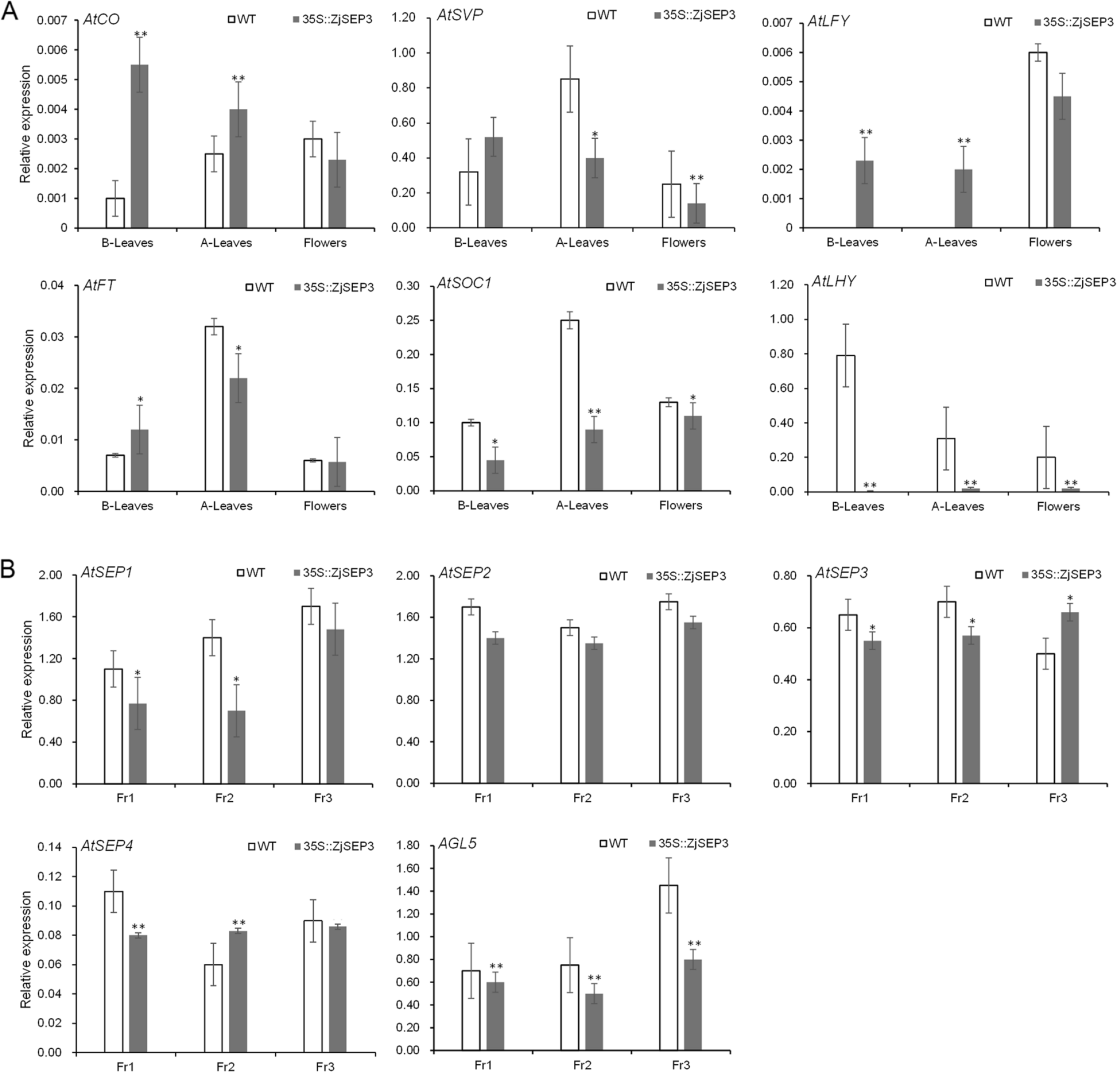
Expression of genes related to flower and fruit development. **A** Expression of genes related to flower development in WT and transgenic plants. ‘B-’ in the x-axis means ‘before flowering’ and ‘A-’ means ‘after flowering’. **B** Expression of genes related to fruit development in WT and transgenic plants. Fr1–3: Fruit development stages 1–3. * represents significantly different from the WT plants at P < 0.05 level and ** represents significantly different from the WT plants at *P* < 0.01 level

Since ZjSEP3 could affect the flowering time in transgenic Arabidopsis, we investigated the expression of 9 crucial genes related to flower development in WT and 35S::*ZjSEP3* transgenic lines. The expression levels of *CO* and *LFY* significantly increased in the leaves of 35S::*ZjSEP3* transgenic plants compared to the WT plants (Fig. 5A). *LFY* expression was detected in the leaves and flowers of 35S::*ZjSEP3* transgenic plants but only in the flowers of the WT plants (Fig. 5A). SVP encodes a nuclear protein that acts as a floral repressor [37]. *SVP* and *FT* exhibited similar expression patterns, and their expression was lower at the flowering stage in 35S::*ZjSEP3* transgenic plants than in the WT plants (Fig. 5A). The expression of *SUPPRESSOR OF OVEREXPRESSION OF CONSTANS1* (*SOC1*), whose overexpression can promote early flowering, was decreased in transgenic plants (Fig. 5A). Moreover, the *AtSEP2* expression were not significant different while *AGL5* expression were depressed in transgenic plants. *AtSEP1* and *AtSEP3* expression were depressed at Fr1 and Fr2 stages (Fig. 5B, Fig. S2).

Notably, *AtLHY* transcription in the leaves and flowers of 35S::*ZjSEP3* transgenic plants was almost totally suppressed at before and after flowering stages (Fig. 5A), indicating that *AtLHY* expression was regulated by *ZjSEP3* in the transgenic plants.

### ZjSEP3 interacts with the CArG-box motifs of the *LHY* promoter

**Fig. 6:**
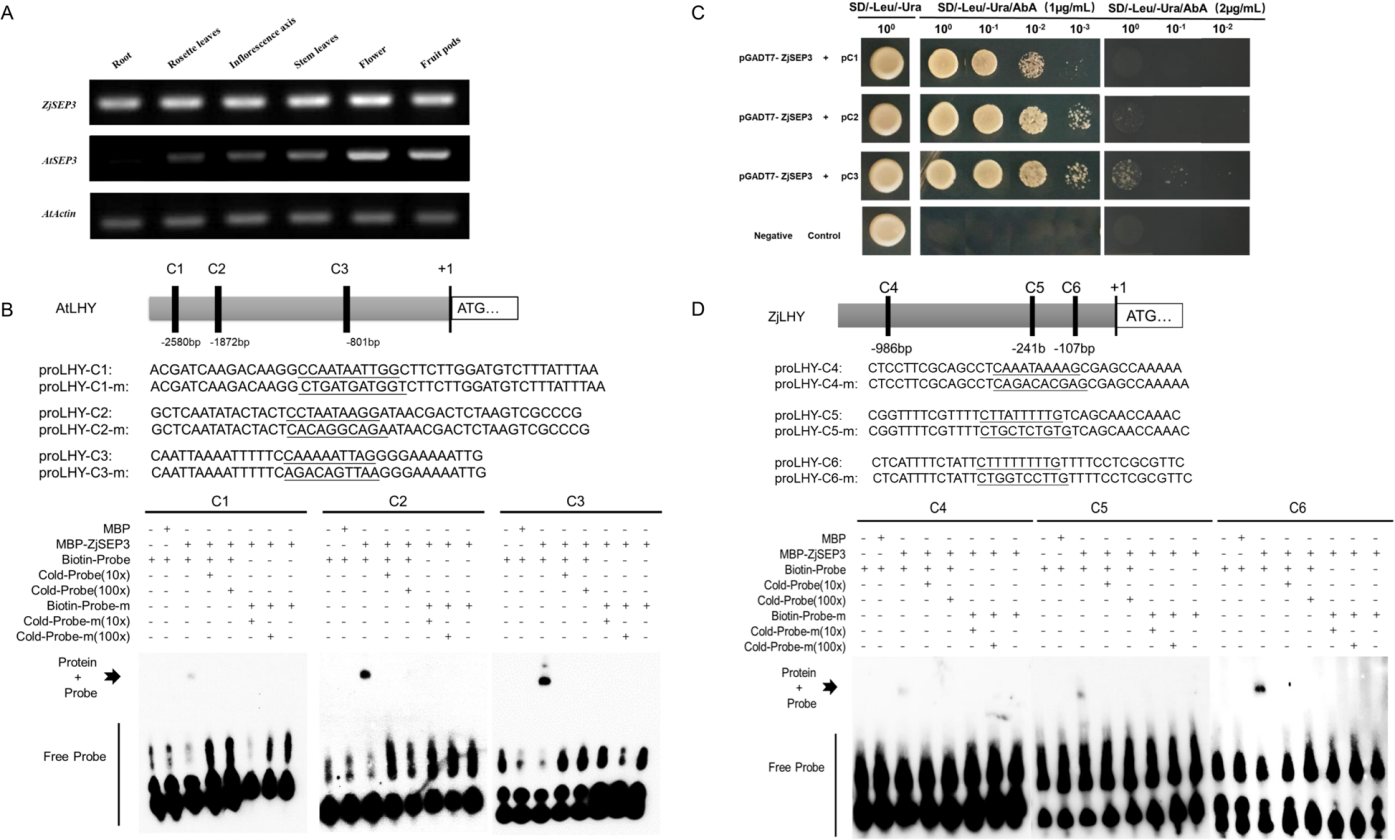
ZjSEP3 interacts with the CArG-box motifs of the AtLHY and ZjLHY promoters. **A** Expression of *ZjSEP3* and *AtSEP3* in 35S::ZjSEP3 transgenic plants. **B** ZjSEP3 binds to the promoter of AtLHY by EMSA. The oligonucleotides (proLHY-C1/2/3 and proLHY-C1/2/3-m) were used as probes. The DNA sequences of proLHY-C2 and proLHY-C3 encode the motifs CC[A/T]_6_GG and C[A/T]_8_G, respectively, termed the CArG-box. The DNA sequence of proLHY-C1 is a highly similar sequence of CArG-boxes. Biotin-Probe-m is biotin-labeled probe with some nucleic acid mutations. MBP, MBP-ZjSEP3, biotin probe, labeled mutated probe (Biotin-Probe-m) and unlabeled probe (Cold-Probe) at 10x and 100x molar excess were present (+) or absent (−) in each reaction. **C** Interaction of full-length ZjSEP3 with different fragments of the AtLHY promoter. pC1, pC2 and pC3, the different CG-box of LHY promoter, were used as the reporter for Y1H; pGADT7-ZjSEP3 was used as the effector of Y1H. Transformed yeast cells containing both effector and reporter were plated on the selective medium SD/−Leu/−Ura/AbA. AbA, Aureobasidin A. pGADT-7 and pAbAi transformation yeast cells were used as negative control. **D** ZjSEP3 binds to the promoter of ZjLHY by EMSA

We tested the expression of *ZjSEP3* and *AtSEP3* in various organs of 35S::*ZjSEP3* transgenic lines. The results showed that *ZjSEP3* was strongly expressed in various organs, including the inflorescence axis, while *AtSEP3* was expressed mainly in the flowers and siliques (Fig. 6A). Previous studies have shown that *AtLHY* is also expressed along the inflorescence axis [38, 39].

Plant MADS-box proteins can bind to specific DNA sequences known as CArG elements [C(C/T)(A/T)6(A/G)G, C(A/T)8G and C(C/T)(A/T)G(A/T)4(A/G)G] [40]. Hence, we searched the promoter sequence of *AtLHY* and identified two CArG-box elements and one similar CArG-box element (Fig. 6B). The presence of these elements indicated that the early flowering of *ZjSEP3* transgenic plants may be caused by the interaction of ZjSEP3 with the *LHY* promoter.

To examine whether *LHY* is a candidate target of ZjSEP3, we performed an EMSA in conjunction with MBP-ZjSEP3 recombinant proteins (Fig. 6B). As shown in Fig. 6B, ZjSEP3 strongly interacted with the *AtLHY* promoter when the primer combinations containing either C2 or C3 were applied, while weak interaction was found between ZjSEP3 and *LHY* promoter containing C1. The result demonstrated that ZjSEP3 can bind to two CArG-box motifs of the AtLHY promoter nearing the coding region, indicating regulation of *AtLHY* transcription by ZjSEP3.

We also performed Y1H assay to investigate the interaction between ZjSEP3 and *AtLHY* promoter. The effector pGADT7-ZjSEP3 and reporters pC1, pC2 and pC3 were co-transformed into Y1H gold yeast respectively because all of them could growing on the SD medium that lacked leucine and uracil (SD/−Leu/−Ura) (Fig. 6C). ZjSEP3 significantly activated AbA resistance in pC1, pC2 and pC3 (Fig. 6C) and obviously pGADT7-ZjSEP3 and pC3 co-transformation yeast showed activation at a higher concentration of AbA (2 μg/mL). Both EMSA and Y1H assays validated that ZjSEP3 binds to C2 and C3 in the promoter region of *AtLHY*.

Moreover, six CArG-box elements were identified in the promoter of ZjLHY and three ones (C4, C5 and C6) were choosing to perform EMSA tests (Fig. 6D). The results further confirmed that ZjSEP3 was capable of binding to the *ZjLHY* promoter.

### Overexpression of *AtLHY* rescues the early-flowering phenotype of 35S::*ZjSEP3* transgenic plants

**Fig. 7:**
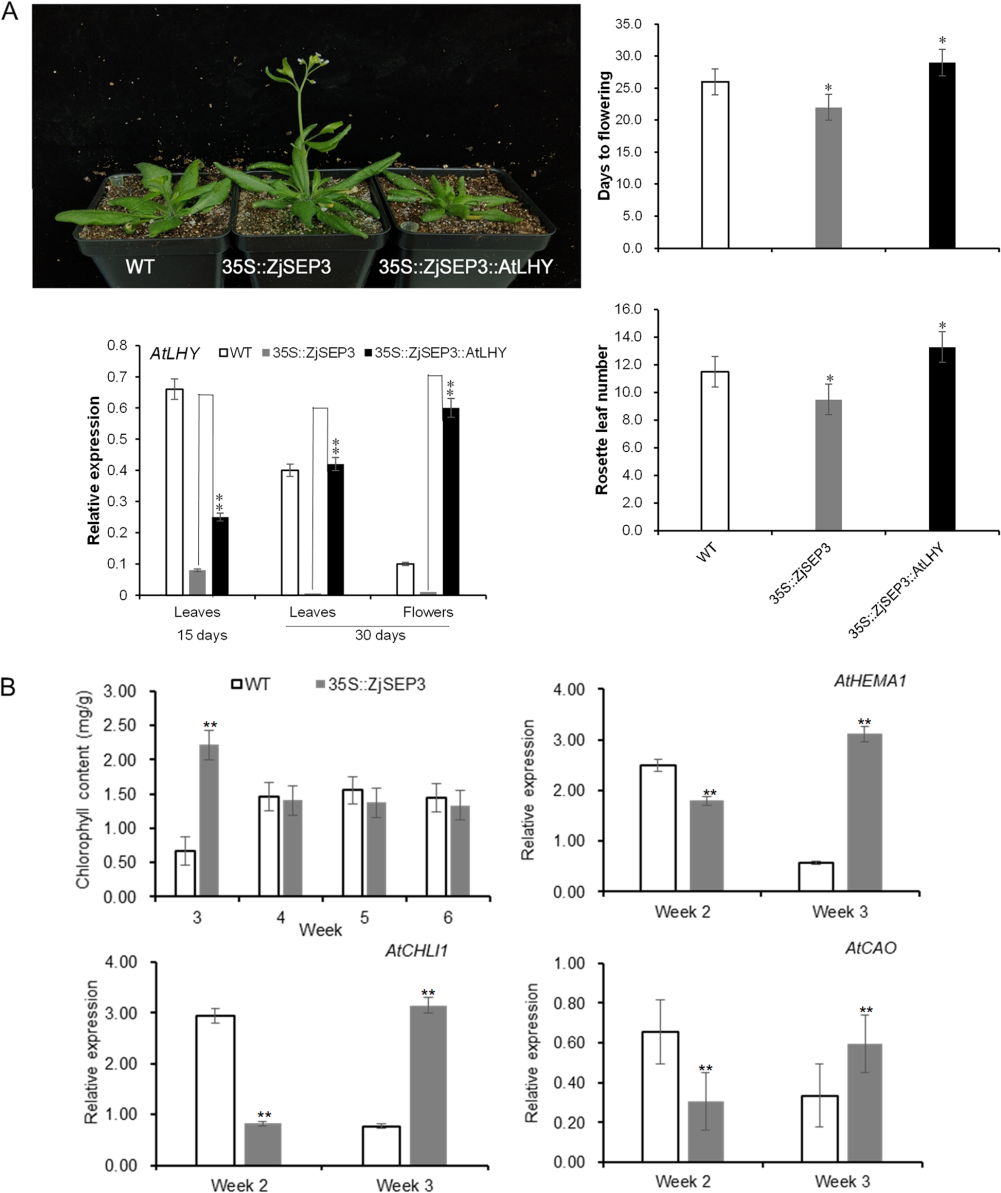
ZjSEP3 promotes chlorophyll synthesis during vegetative growth stages. **A** Overexpression of AtLHY rescues the early-flowering phenotype of 35S::ZjSEP3 transgenic plants. AtLHY expression, the days to flowering and the number of rosette leaves of WT, 35S::ZjSEP3 and 35S::ZjSEP3/35S::AtLHY plants were compared. **B** Chlorophyll content in the rosette leaves of wild-type and 35S::ZjSEP3 transgenic Arabidopsis plants. Expression profiles of three genes associated with chlorophyll synthesis. Week-2 leaves: leaves were sampled before flowering. Week-3 leaves: leaves were sampled after flowering. * represents significantly different from the WT plants at P < 0.05 level and ** represents significantly different from the WT plants at P < 0.01 level

To further demonstrate the regulation of *AtLHY* by ZjSEP3, *AtLHY* was overexpressed in 35S::*ZjSEP3* transgenic plants and then observed their flowering phenotypes (Fig. 7A). As shown in Fig. 7A, the flowering times were similar between 35S::*ZjSEP3*/35S::*AtLHY* transgenic plants and WT plants, and compared with that in the 35S::*ZjSEP3* plants, the expression of *AtLHY* in the 35S::*ZjSEP3*/35S::*AtLHY* transgenic plants increased significantly. The results confirmed that overexpression of *AtLHY* rescues the early- flowering phenotype of 35S::*ZjSEP3* transgenic plants. Thus, both the phenotypic analysis and molecular evidence indicated that ZjSEP3 acts upstream of *LHY* and functions as a positive regulator of flowering time.

### *ZjSEP3* promotes chlorophyll synthesis during the vegetative growth stages

ZjSEP3 acted upstream of *AtLHY*, which plays crucial roles in the maintenance of the circadian rhythm [33, 35, 41], meaning that *ZjSEP3* should influence a series of rhythmic growth. The rosette leaves of 35S::*ZjSEP3* transgenic plants were dark green, so their chlorophyll content was subsequently measured. The results showed that the chlorophyll content of the rosette leaves of 35S::*ZjSEP3* seedlings (week 3) was significantly higher than that of the WT seedlings (Fig. 7B). Compared to WT Arabidopsis, the expression of the key genes (*HEMA1*, *CHLI1* and *CAO*) involved in the chlorophyll synthesis in 35S::*ZjSEP3* plants was significantly increased at the early reproductive stages (Fig. 7B). *Schaffer* et al. also showed that a gain-of-function *lhy* mutation caused an elongated hypocotyl and reduced chlorophyll content [42], indicating that *LHY* transcription might involve in chlorophyll synthesis.

In this study, the chlorophyll content of 35S::*ZjSEP3* seedlings, in which the *AtLHY* transcription was obviously suppressed, was significantly higher than that of the WT seedlings. These results might reveal that ectopic expression of *ZjSEP3* in Arabidopsis could alter rhythmic growth by modulating of *LHY* and ultimately promote flowering via rapid growth and early nutrient accumulation during the vegetative and early reproductive stages.

## Discussion

*SEP3* plays a crucial role in plant flowering and fruiting [16, 18, 43]. Here, we combined genetic transformation, biochemical, and bioinformatic analyses to provide evidence for the function of ZjSEP3 in the regulation of flowering time by binding to *LHY* promoter. In Arabidopsis, SEP3 acts the direct or indirect downstream gene of *LFY*, *SVP*, *SOC* and *FT* [39, 44]. However, in this study overexpression of *ZjSEP3* leads to various changes in their expression. These results were not contradictory, because the changes of these genes were not induced by *ZjSEP3* directly. Instead, *ZjSEP3* modulated their expression by regulating *LHY*, which is an upstream regulator of these genes. However, the in vivo binding of SEP3 to LHY genomic region was not found in a previous study [18].

As one of the MADS-box transcription factors, SEP3 is not the first report to interact with *LHY* promoter. Spensley et al. showed that the MADS-box transcription factor FLOWERING LOCUS C (FLC) can also bind to the *LHY* promoter and the interaction was only detected reliably in FLC-overexpressing plants [31]. The ectopic regulations of SEP3 and FLC are both related to CArG boxes, which are bound by MADS-box transcription factors. Thus, further studies will be required to test whether other MADS-box proteins binding to the *LHY* promoter exhibits such regulation.

In 35S::*ZjSEP3* transgenic plants, *AtLHY* expression was significantly suppressed (Fig. 5A). Previous studies reported that *LHY*-defective mutants exhibited accelerated flowering and elongated hypocotyls under both long and short days [25, 42], and their early flowering phenotypes were more prominent under short days. These results were consistent with those of our study. Our work highlights the potential of SEP3 as a positive regulator of flowering time by regulating *LHY*.

Both *LHY* and *CIRCADIAN CLOCK ASSOCIATED 1* (*CCA1*) are MYB-like transcription factors and exhibit strong homology in terms of their MYB components [33, 45]. Many individual *LHY* and *CCA1* loss-of-function mutants exhibited phenotypes whose rhythm was truncated [25, 35, 46, 47]. However, these genes’ expressions behaved differently in 35S::*ZjSEP3* Arabidopsis (Fig. 5, Fig. S3), meaning that they should have distinct regulatory pathways or roles in the regulation of flowering. Our results showed that ZjSEP3 could strongly bind to the CArG-box region within the *LHY* promoter (Fig. 6B, C, D), while the CArG-box or similar regions were not found within the *CCA1* promoter. Park et al. also reported that CCA1 can bind strongly to the *FT* promoter but that LHY does not exhibit such binding activity [42]. These different binding regions likely explain why ZjSEP3 could inhibit the transcription of *LHY* but not *CCA1*. The above results are consistent with those of the study showing that *LHY* and *CCA1* have different biochemical activities [48].

Here, a series of biochemical and genetic experiments demonstrated that ZjSEP3 bound to the *AtLHY* promoter and inhibited its expression. We were interested in whether the binding role of this protein occurred in other plant species. Therefore, we searched the jujube, pear, apple, peach and mulberry genome sequences and found that all of them contained two or more CArG-boxes within the promoter of the *LHY* gene (Fig. S4). As shown in Fig. S4, most of CArG-boxes were located nearing the coding region, indicating that this regulation should exist in Chinese jujube and other Rosa species when overexpression of *ZjSEP3* in these plants. At present, the genetic transformation system in Chinese jujube is not successful, so we can only verify its function in Arabidopsis.

Using Y2H and BiFC assays, we further investigated whether ZjSEP could interact with AtLHY at the protein level. The results clearly showed that the ZjSEP3 protein could interact with the AtLHY protein (Fig. S5A) and the interacting protein complex appeared to be located in the nucleus (Fig. S5B). In addition to Arabidopsis and jujube, the interaction was also observed in other three Rosaceae species (apple, pear and peach) (Fig. S5A). These results suggested that ZjSEP3 could regulate LHYs at transcription and translation levels, and this multi-level regulation is also found in other transcription factors [21].

Notably, *Populus* trees were recently used to study the function of *LHY* on the growth of perennial species [49]. *lhy-10* trees, whose *LHY1* and *LHY2* expression was reduced by RNAi, exhibited a short free-running period and an increased lignification zone in the stem, and their genes important for growth regulation were expressed at an earlier phase. The results indicated that the function of *LHY* in perennial species was similar to that in annual species.

Shortening the juvenile phase and promoting flowering time in perennial fruit trees is very beneficial, especially in terms of breeding. Early flowering of perennial fruit trees could not only save time and labor in breeding but also promote earlier fruit production. Previous studies have shown that Chinese jujube is closely related to Rosaceae species [22]. Rosaceae species such as apple, pear and peach have a longer juvenile phase (approximately 3–5 years) than does jujube (only 1–2 years), and the above results indicated that, by regulating *LHY*, overexpression of *ZjSEP3* might accelerate the flowering time of those perennial species. It is worth noting that there are three typical CArG-boxes within the range of upstream 1000 bp of *ZjLHY* (Fig. S4), and it also confirmed to be regulated by ZjSEP3 (Fig. 6D). Although the transgenic technique is not successful in Chinese jujube and some other perennial trees, the results of the present study provide a strong potential gene for transgenic breeding of fruit trees and reveal a novel regulatory network involving *SEP3* and *LHY*.

## Conclusions

*ZjSEP3* transgenic Arabidopsis exhibits an early flowering phenotype, in which the expression of the Arabidopsis biorhythm-related gene *LHY* is significantly suppressed. It found that ZjSEP3 can interact with both *AtLHY* and *ZjLHY* upstream promoter CArG-box sequences. Furthermore, overexpression of *AtLHY* in *ZjSEP3* transgenic Arabidopsis can compensate for the early flowering phenotype. These results suggest that SEP3 acts as a positive regulator of flowering time by regulating the expression of *LHY*.

## Materials and methods

### Materials

Four cultivars of *Z. jujuba* (‘Dongzao’, ‘Hongzhenzhu’, ‘JMS1’ and ‘JMS2’) were obtained from the Experimental Station of Chinese Jujube, Hebei Agricultural University. Four kinds of flower organs (sepals, petals, stamens and pistils) were sampled in May 2017, and flowers at six development stages were also collected. Three independent biological replicates were included for all treatments. All samples were immediately frozen in liquid nitrogen and stored at − 80 °C until they were used.

### Phylogenetic analysis of SEP3 in different plant species

The SEP sequences of other species aside from jujube were obtained from the NCBI database (http://www.ncbi.nlm.nih.gov/genbank/) (Table S1). We used ClustalX 2.0 to perform multiple protein sequence alignments of ZjSEP3 and other E-type proteins from different plant species. A phylogenetic tree was generated using the neighbor-joining method and a bootstrap confidence test (1000 replicates). CRM6 from *Ceratopteris pteridoides* and DAL1 from *Picea abies* were used as out groups.

### Quantitative real-time PCR (qRT-PCR) analysis

Total RNA from jujube and Arabidopsis plants was isolated using an RNA extraction kit (Tiangen, China). The RNA was then reverse transcribed by AMV reverse transcriptase (Takara, Japan) according to the manufacturer’s instructions. The relative expression of genes was subsequently analyzed by qRT-PCR, which was performed using gene-specific primers (Table S2). Three biological replications were performed for each experiment. Each qRT-PCR mixture (20 μL) comprised 1 μL of cDNA, 0.4 μL of each primer, 8.2 μL of ddH_2_O and 10 μL of 2× TransStart Top Green qPCR SuperMix. The PCR program was as follows: 95 °C for 15 min, followed by 40 cycles of 95 °C for 10 s, 58 °C for 35 s, and 72 °C for 35 s. The fluorescence signal was measured at 55 s. *ZjACT* and *AtACT* were used as internal controls, respectively. The gene expression values were normalized against the expression value of the reference gene in accordance with the 2^–ΔCT^ method.

### Identification and cloning of *LHY* genes

Total RNA was isolated from Chinese jujube (*Z. jujuba*), apple (*Malus* × *domestica*), peach (*P. persica*) and pear (*Pyrus* × *bretschneideri*) by using an RNAprep Pure Plant Kit (Polysaccharides & Polyphenolics-rich; Tiangen, China) in accordance with the manufacturer’s instructions. The gene-specific primers were designed according to the accession numbers XM_016033463.2, XM_008345245.2, XM_007218867.2 and XM_01864 2751.1 (Table S2). We used a TIANScript First Strand cDNA Synthesis Kit (Tiangen, China) to synthesize first-strand complementary DNA (cDNA). Full-length cDNA was obtained by performing PCR in a 50 μL volume that comprised 0.25 μL of SpeedSTAR HS DNA Polymerase (TaKaRa, China), 4 μL of a dNTP mixture (2.5 mM), each primer at 10 μM, 2 μL of cDNA, and 5 μL of 10× Fast Buffer I (Mg^2+^ plus), with ddH_2_O added to reach final volume of 50 μL. The PCR amplification procedure was as follows: 5 min at 95 °C; 35 cycles of 8 s at 98 °C, 20 s at 60 °C, and 20 s at 72 °C; and 10 min at 72 °C. The PCR product was preserved at 4 °C. All target fragments were cloned into a pMD19-T vector (TaKaRa, China) using Solution I (TaKaRa, China) and then transformed to DH5а (Tiangen, China).

### Subcellular localization and transcriptional function assays

The open reading frame (ORF) of *ZjSEP3* was amplified and cloned into a pCAM-GFP vector in frame with the GFP gene. The resulting plasmids were introduced into *Agrobacterium tumefaciens* (strain GV3101), which were then injected into *Nicotiana benthamiana* leaves. The infected tissues were evaluated 48 h after infiltration. The tobacco plants were grown at 22 °C under 16 h light/8 h dark conditions.

The ORF of *ZjSEP3* was cloned into pGBKT7, and the resulting recombinant plasmids were transformed into yeast strain AH109. Successful transformants were selected on synthetically defined (SD) medium that lacked tryptophan (SD-Trp). Yeast selective media (SD-Trp-His-Ade) were used to evaluate transcriptional function. The individual yeast colonies were then transferred to amicrobic filter paper, after which they were subjected to repeated freezing and thawing in liquid nitrogen to disrupt the cells. Last, the filter paper was soaked with X-gal/Z-buffer solution, and the colonies were cultured at 28 °C in the dark to observe the color. X-gal/Z-buffer solution: 100 mL of Z-buffer, 0.27 mL of β-mercaptoethanol, and 1.67 mL of X-gal. Z-buffer solution: 16.1 g/L Na_2_HPO_4_•7H_2_O, 5.50 g/L NaH_2_PO_4_•H_2_O, 0.75 g/L KCl, 0.246 g/L MgSO_4_•7H_2_O, pH 7.0, 20 mg/mL X-gal (DMF dissolution). BD-empty constructs served as negative controls.

### Arabidopsis growth and genetic transformation

Under the control of the 35S promoter, *ZjSEP3* was cloned into pSN1301 vectors for construction of overexpression plasmids. *A. tumefaciens* strain GV3101 was transformed with the construct, and *Arabidopsis thaliana* plants were transformed using the floral dip method. First-generation seeds of the *ZjSEP3* transgenic plants were selected via Murashige and Skoog (MS) medium supplemented with hygromycin B (50 mg/L). The transgenic plants were confirmed by using real-time PCR (RT-PCR), and the homozygotic lines were selected for three or more generations.

Under the control of the 35S promoter, the *AtLHY* gene was cloned into a pCAMBIA3301 plasmid vector. *A. tumefaciens* strain GV3101 was then transformed with the construct, and the *35S::ZjSEP3* transgenic lines were transformed using the floral dip method. First-generation seeds of the *ZjSEP3* and *AtLHY* co-transgenic plants were selected via MS medium supplemented with 10% Basta solution (40 μL/L). The transgenic plants were confirmed by RT-PCR, and the homozygotic lines were selected for three or more generations.

Arabidopsis wild-type and transgenic plants were maintained at 22 °C under a 16 h light/8 h dark photoperiod in a growth chamber. To ascertain the sampling time, the diurnal expressions of *AtLHY* in WT and 35S::*ZjSEP3* Arabidopsis plants were analyzed in advance by qRT-PCR depending on Zeitgeber Time (ZT, Fig. S6). The leaves of Arabidopsis plants were collected every 4 h for the time course experiment. Three biological replicates were used for each time point. In the following experiments, the samples were collected at ZT2 for the expression analysis of the flower-related genes. The flowering time of the Arabidopsis seedlings was recorded by scoring the number of rosette leaves when the primary inflorescence was 1.0 cm long. Thirty Arabidopsis plants in each treatment were scored. The rosette leaves and flowers were sampled at various stages (before and after flowering) to evaluate gene expression.

### Electrophoretic mobility shift assays (EMSAs)

EMSAs were conducted using a LightShift™ EMSA Optimization & Control Kit (Thermo Prod#20148X) and a Chemiluminescent Nucleic Acid Detection Module (Thermo 89,880) in accordance with the manufacturer’s protocol. The recombinant maltose-binding protein (MBP)-ZjSEP3 protein and MBP protein were purified from *E. coli* BL21 using MBP beads (New England BioLabs). The DNA fragments and mutated sequences of the LHY promoter were synthesized and labeled with biotin at the 5′ DNA terminus for serving as biotin probes. Biotin-unlabeled fragments of the same sequences or mutated sequences were used as cold probes. MBP alone was used as the negative control.

### Yeast one-hybrid (Y1H)

Yeast one-hybrid assay were used to investigate the interaction of ZjSEP3 with its supposed target gene promoter. According to EMSA assay results, we choose three DNA fragments of *AtLHY* promoter pC1, pC2 and pC3 consisting three CG-box respectively. The sequence of pC1, pC2 and pC3 were shown in Table S3. The three DNA fragments were connected into the pAbAi vector respectively as the reporters of Y1H. The full length cDNA of *ZjSEP3* was cloned into the pGADT7 vector containing a GAL4 transcriptional domain as the effector of Y1H. Effector and each of those reporters were co-transformed into Y1H gold yeast strain and then selected on SD medium that lacked leucine and uracil (SD/−Leu/−Ura). Aureobasidin A (AbA) was used for evaluating the interactions between SEP3 and *LHY* promoters.

### Yeast two-hybrid (Y2H)

A Matchmaker GAL4 Two-Hybrid System 3 kit was used for Y2H assays. Activation domain (AD)-fused MADS-box genes and binding domain (BD)-fused MADS-box genes were amplified using the primers shown in Table S2 and then cloned into pGADT7 and pGBKT7 vectors, respectively. All the MADS-box genes were digested by EcoR I and then cotransformed with pairs of appropriate pGADT7 and pGBKT7 vectors. Successful cotransformants were selected on SD medium that lacked tryptophan and leucine (SD/−Trp/ -Leu). Three selective media, tryptophan/leucine/histidine-lacking SD (SD/−Trp/−Leu/−His), SD/−Trp/−Leu/−His containing 5 mM 3-amino-1,2,4-triazole (SD/−Trp/Leu/−His+3AT), and tryptophan/leucine/histidine/adenine-lacking SD (SD/−Trp/−Leu/−His/−Ade), were used for evaluating the protein interactions.

### Bimolecular fluorescence complementation (BiFC) assays

With respect to the BiFC assays, the full-length *ZjSEP3* and *AtLHY* sequences were cloned into pSPYNE and pSPYCE vectors, respectively. The resulting plasmids were introduced into *A. tumefaciens* (strain GV3101), which were then injected into *N. benthamiana* leaves. Infected tissues were observed at 48 h after infiltration. The primers used for BiFC are listed in Table S2.

### Determination of chlorophyll content

Arabidopsis leaves (0.1 g) were cut into small pieces and dipped into 95% ethyl alcohol in the dark for 24 h to extract chlorophyll. The absorbance at 665 nm and 649 nm was determined using a spectrophotometer (UNICO® UV/VIS 2802PC). The formulas were as follows: Ca = 13.95OD_665_–6.88OD_649_; Cb = 24.95OD_649_–7.32OD_665_; total Chl (mg/g) = Ca/Cb × volume (L) × times diluted/ dry weight (g).

### Statistical analysis

The data in this study are presented as the means ± SDs of at least three independent experiments. Statistical analyses were conducted using the one-way ANOVA test with Excel software (Microsoft Office, 2010). Statistically significant differences were indicated either with * (*P* < 0.05) or with ** (*P* < 0.01).

## Supplementary Information

**Additional file 1: Figure S1**. The growth conditions of WT and 35S::ZjSEP3 Arabidopsis plants at four-leaf stage. These values of two independent different transgenic lines were provided, and 30 plants were measured in each line.

**Additional file 2: Figure S2**. The siliques of WT and 35S::ZjSEP3 Arabidopsis plants at three stages (Fr1, Fr2, Fr3).

**Additional file 3: Figure S3**. *AtCCA1* expression in wild-type and transgenic Arabidopsis. ‘B-’ in the x-axis means ‘before flowering’ and ‘A-’ means ‘after flowering’.

**Additional file 4: Figure S4**. The CArG-boxes within the LHY promoter in various plant species. Note: *LHY* promoters of jujube (*ZjLHY*, *Ziziphus jujuba*, XM_016033463.2), apple (*MdLHY*, *Malus* × *domestica*, XM_008345245.2), peach (*PpLHY*, *Prunus persica*, XM_007218867.2), and pear (*PbLHY*, *Pyrus* × *bretschneideri*, XM_018642751.1) as well as mulberry (*MnLHY, Morus notabilis*, XM_024172697.1) are shown in the figure. The binding sequences include the following: C1 (CTAATTAATG), C2 (CATGAAAAAG), C3 (CTTTTTTATG), C4 (CAAATAAAAG), C5 (CTTATTTTTG), C6 (CTTTTTTTTG), C7 (CAAA TTTATG), C8 (CCAGAAATGG), C9 (CTAAAAAAAG), C10 (CTAAATTTTG), C11 (CTTTTTTT AG), C12 (CTATATTAAG), C13 (CCAAAAATAG), C14 (CAATTTATTG), C15 (CTATTTAAAG) and C16 (CATTTTTTAG).

**Additional file 5: Figure S5**. ZjSEP3 interacts with LHYs of various species. (A) *LHYs* fused to the GAL4 AD were expressed in combination with *ZjSEP3* fused to the GAL4 DNA-BD in yeast strain AH109. The negative controls included the following: (1) BD-fused SEP3 co-expressed with empty ADs and (2) AD-fused LHYs co-expressed with empty BDs. Yeast cells harboring AD and BD vectors were adjusted to an optical density at 600 nm (OD600) of 0.1. Aliquots (10 μL) of these cells were spotted on selective medium that lacked leucine/tryptophan (−LW), leucine/tryptophan/histidine (−LWH) and tryptophan/leucine/adenine/histidine (−LWAH). The plates were incubated for 3–4 days at 30 °C. Yeast cells expressing BD-fused ZjSEP and each of the AD-fused LHYs grew on selective media, while yeast cells expressing empty BD- and AD-fused LHYs did not grow. ZjMADS46, a C/D class protein of Chinese jujube, was used as positive control. (B) BiFC assay of the interaction between ZjSEP3 and AtLHY in agro-infiltrated *Nicotiana benthamiana* leaves. CYFP: C-terminus of YFP; NYFP: N-terminus of YFP; ZjSEP3-NYFP: ZjSEP3 fused to the N- terminus of YFP; AtLHY-CYFP: AtLHY fused to the C-terminus of YFP; Yellow: Yellow fluorescent protein (YFP) fluorescence. The interaction of ZjSEP3-NYFP with CYFP and NYFP with AtLHY-CYFP, respectively, are shown as negative controls. No signals of interactions were observed from ZjSEP3-NYFP + CYFP and NYFP + AtLHY-CYFP. Yellow fluorescent BiFC signals were detected from ZjSEP3-NYFP + AtLHY-CYFP, suggesting that *ZjSEP3* strongly interacted with AtLHY in the nucleus.

**Additional file 6: Figure S6**. The diurnal expression patterns of AtLHY in WT and 35S::*ZjSEP3* Arabidopsis. White and black bars represent light and dark periods, respectively.

**Additional file 7: Table S1**. NCBI reference of the genes in this study.

**Additional file 8: Table S2**. Information on the primers listed in this study.

**Additional file 9: Table S3**. DNA fragments used in Y1H.

## Data Availability

All data and materials are presented in the main paper and additional supporting file.

## Abbreviations

WT: Wild-type Columbia Arabidopsis
SEP: SEPALLATA
LHY: LATE ELONGATED HYPOCOTYL
Y1H: Yeast one-hybrid
Y2H: Yeast two-hybrid
qRT-PCR: Quantitative real- time PCR analysis
EMSAs: Electrophoretic mobility shift assays
GFP: Green fluorescent protein
BiFC: Bimolecular fluorescence complementation assays
