# Supplementary figures and images for: ZjSEP3 modulates flowering time by regulating the LHY promoter
Source: BMC Plant Biol. 2021 Nov 11;21:527. doi: 10.1186/s12870-021-03305-x (PMC8582215; doi:10.1186/s12870-021-03305-x)

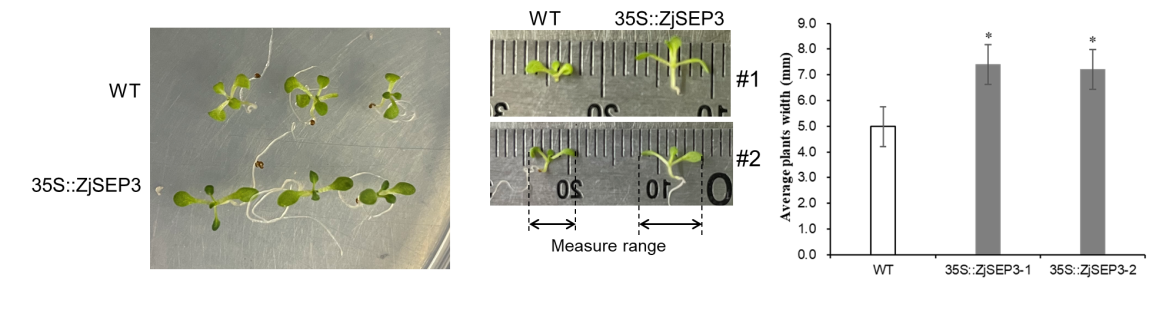

Supplement: Supplementary file 1 — Additional file 1: Figure S1. The growth conditions of WT and 35S::ZjSEP3 Arabidopsis plants at four-leaf stage. These values of two independent different transgenic lines were provided, and 30 plants were measured in each line. [file 12870_2021_3305_MOESM1_ESM.docx]

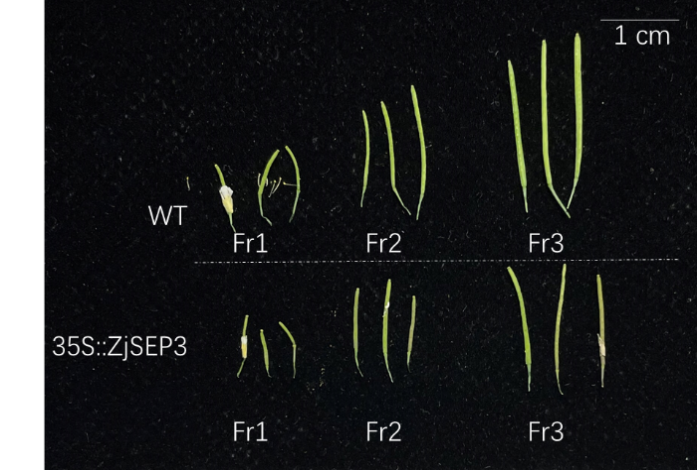

Supplement: Supplementary file 2 — Additional file 2: Figure S2. The siliques of WT and 35S::ZjSEP3 Arabidopsis plants at three stages (Fr1, Fr2, Fr3). [file 12870_2021_3305_MOESM2_ESM.docx]

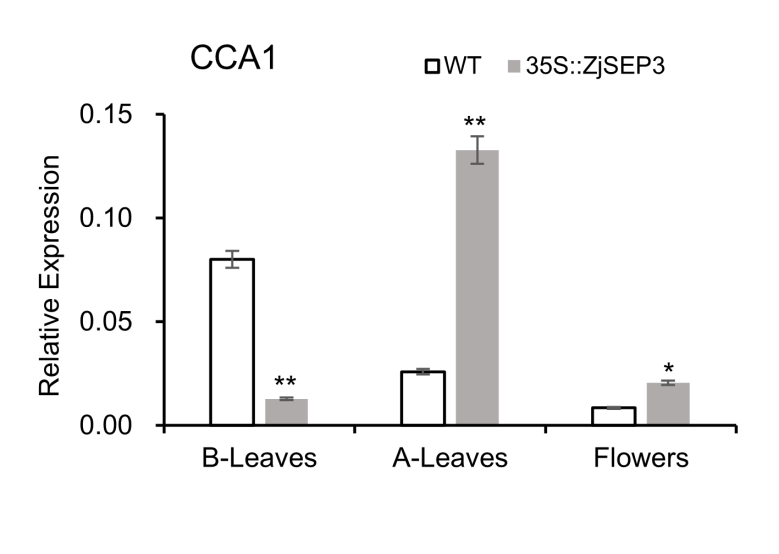

Supplement: Supplementary file 3 — Additional file 3: Figure S3. AtCCA1 expression in wild-type and transgenic Arabidopsis. ‘B-’ in the x-axis means ‘before flowering’ and ‘A-’ means ‘after flowering’. [file 12870_2021_3305_MOESM3_ESM.docx]

**
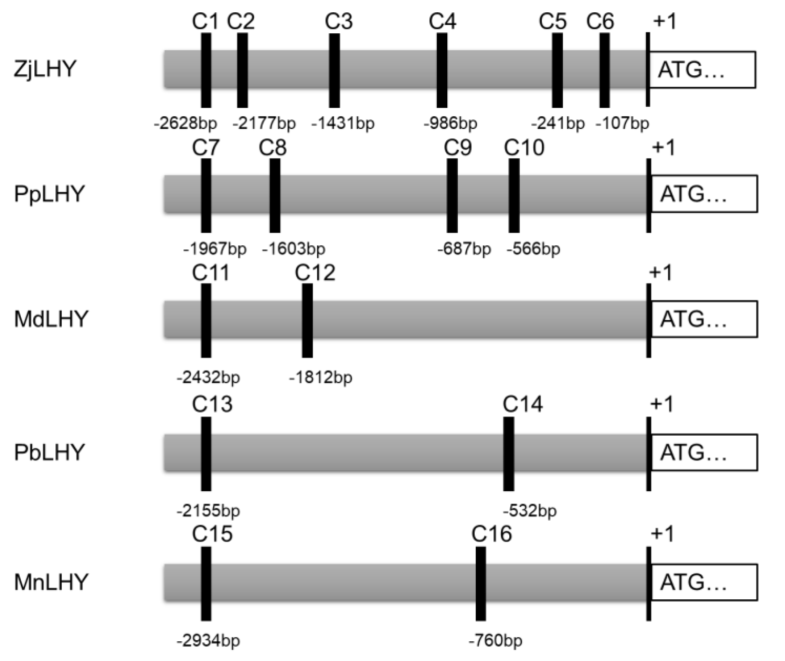
**

Supplement: Supplementary file 4 — Additional file 4: Figure S4. The CArG-boxes within the LHY promoter in various plant species. Note: LHY promoters of jujube (ZjLHY, Ziziphus jujuba, XM_016033463.2), apple (MdLHY, Malus × domestica, XM_008345245.2), peach (PpLHY, Prunus persica, XM_007218867.2), and pear (PbLHY, Pyrus × bretschneideri, XM_018642751.1) as well as mulberry (MnLHY, Morus notabilis, XM_024172697.1) are shown in the figure. The binding sequences include the following: C1 (CTAATTAATG), C2 (CATGAAAAAG), C3 (CTTTTTTATG), C4 (CAAATAAAAG), C5 (CTTATTTTTG), C6 (CTTTTTTTTG), C7 (CAAA TTTATG), C8 (CCAGAAATGG), C9 (CTAAAAAAAG), C10 (CTAAATTTTG), C11 (CTTTTTTT AG), C12 (CTATATTAAG), C13 (CCAAAAATAG), C14 (CAATTTATTG), C15 (CTATTTAAAG) and C16 (CATTTTTTAG). [file 12870_2021_3305_MOESM4_ESM.docx]

**
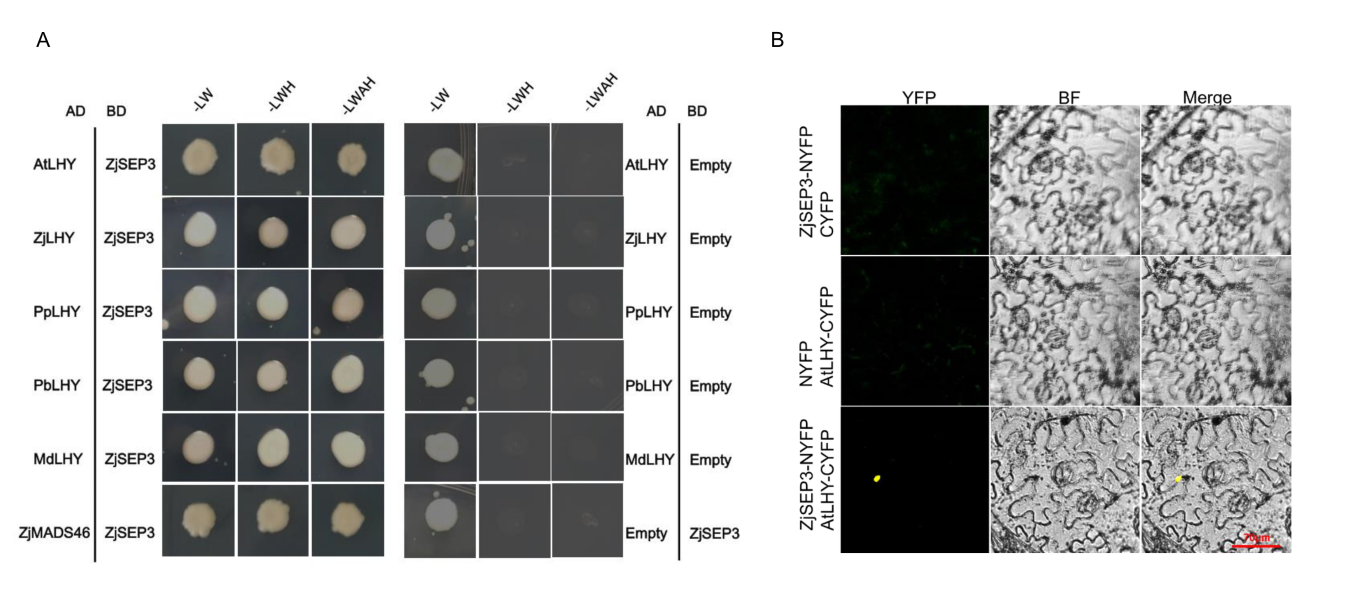
**

Supplement: Supplementary file 5 — Additional file 5: Figure S5. ZjSEP3 interacts with LHYs of various species. (A) LHYs fused to the GAL4 AD were expressed in combination with ZjSEP3 fused to the GAL4 DNA-BD in yeast strain AH109. The negative controls included the following: (1) BD-fused SEP3 co-expressed with empty ADs and (2) AD-fused LHYs co-expressed with empty BDs. Yeast cells harboring AD and BD vectors were adjusted to an optical density at 600 nm (OD600) of 0.1. Aliquots (10 μL) of these cells were spotted on selective medium that lacked leucine/tryptophan (−LW), leucine/tryptophan/histidine (−LWH) and tryptophan/leucine/adenine/histidine (−LWAH). The plates were incubated for 3–4 days at 30 °C. Yeast cells expressing BD-fused ZjSEP and each of the AD-fused LHYs grew on selective media, while yeast cells expressing empty BD- and AD-fused LHYs did not grow. ZjMADS46, a C/D class protein of Chinese jujube, was used as positive control. (B) BiFC assay of the interaction between ZjSEP3 and AtLHY in agro-infiltrated Nicotiana benthamiana leaves. CYFP: C-terminus of YFP; NYFP: N-terminus of YFP; ZjSEP3-NYFP: ZjSEP3 fused to the N- terminus of YFP; AtLHY-CYFP: AtLHY fused to the C-terminus of YFP; Yellow: Yellow fluorescent protein (YFP) fluorescence. The interaction of ZjSEP3-NYFP with CYFP and NYFP with AtLHY-CYFP, respectively, are shown as negative controls. No signals of interactions were observed from ZjSEP3-NYFP + CYFP and NYFP + AtLHY-CYFP. Yellow fluorescent BiFC signals were detected from ZjSEP3-NYFP + AtLHY-CYFP, suggesting that ZjSEP3 strongly interacted with AtLHY in the nucleus. [file 12870_2021_3305_MOESM5_ESM.docx]

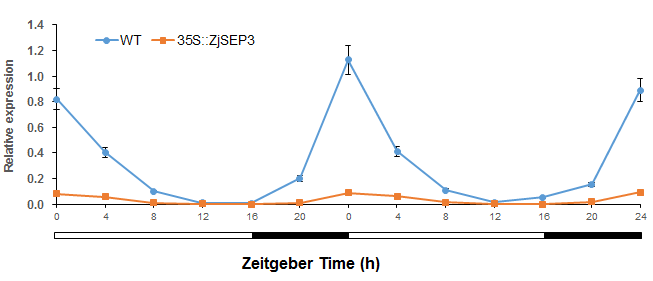

Supplement: Supplementary file 6 — Additional file 6: Figure S6. The diurnal expression patterns of AtLHY in WT and 35S::ZjSEP3 Arabidopsis. White and black bars represent light and dark periods, respectively. [file 12870_2021_3305_MOESM6_ESM.docx]
