# Supplementary material for: ZjSEP3 modulates flowering time by regulating the LHY promoter
Source: BMC Plant Biol. 2021 Nov 11;21:527. doi: 10.1186/s12870-021-03305-x (PMC8582215; doi:10.1186/s12870-021-03305-x)
Supplement: Supplementary file 7 — Additional file 7: Table S1. NCBI reference of the genes in this study. [file 12870_2021_3305_MOESM7_ESM.docx]

| **Table S1 NCBI reference of the genes in this study.** | |
| --- | --- |
| **Gene name** | **NCBI reference** |
| ZjSEP3 | XM_016038815.1 |
| ZjMADS46 | XM_016024828.1 |
| ZjLHY | XM_016033463.2 |
| ZjActin | XM_016021501 |
| AtCAO | AT1G44446.1 |
| AtCHLH | AT5G13630.1 |
| AtHEMA1 | AT1G58290.1 |
| AtFT | AT1G65480.1 |
| AtCO | AT5G15840.1 |
| AtSVP | AT2G22540.1 |
| AtLFY | AT5G61850.1 |
| AtLHY | AT1G01060.1 |
| AtSOC1 | AT2G45660.1 |
| AtActin | AT3G18780.3 |
| PbLHY | XM_018642751.1 |
| PpLHY | XM_007218867.2 |
| MdLHY | XM_008345245.2 |
