# Supplementary material for: ZjSEP3 modulates flowering time by regulating the LHY promoter
Source: BMC Plant Biol. 2021 Nov 11;21:527. doi: 10.1186/s12870-021-03305-x (PMC8582215; doi:10.1186/s12870-021-03305-x)
Supplement: Supplementary file 8 — Additional file 8: Table S2. Information on the primers listed in this study. [file 12870_2021_3305_MOESM8_ESM.docx]

| **Table S2 Information on the primers listed in this study.** | | |
| --- | --- | --- |
| **Gene name** | **Primers(5’-3’)** | **Experiment** |
| BD-SEP3-S | ATGGCCATGGAGGCCGATGGGAAGAGGTAGAGTGGA | Y2H |
| BD-SEP3-A | GGATCCCCGGGAATTTCATGGTAACCATCCTGGCA |  |
| AD-ZjMADS46-S | CCATGGAGGCCAGTGATGGAGTTCCCAAATCAAGCA |  |
| AD-ZjMADS46-A | CACCCGGGTGGAATTTCAAACAAGTTGGAGAGCTG |  |
| AD-ZjLHY-S | CCATGGAGGCCAGTGATGGAGGCATATTCCTCCGG |  |
| AD-ZjLHY-A | CACCCGGGTGGAATTTCAAGTTGAAGCTTCCCCT |  |
| AD-PbLHY-S | CCATGGAGGCCAGTGATGGGACAATCAATTGACA |  |
| AD-PbLHY-A | CACCCGGGTGGAATTTCAAGTTTGAGCTTCCCCTTC |  |
| AD-PpLHY-S | CCATGGAGGCCAGTGATGGAAAGTTGGAAACAACTTTC |  |
| AD-PpLHY-A | CACCCGGGTGGAATTTCAAGTTTGAGCTTCCCCTTC |  |
| AD-MdLHY-S | CCATGGAGGCCAGTGATGGACACAAACTCATCTGG |  |
| AD-MdLHY-A | CACCCGGGTGGAATTTCAAGTTTGAGCTTCCCCTTC |  |
| q-ZjSEP3-S | GGGGAAGACCTTGGACCTTT | q-PCR |
| q-ZjSEP3-A | AGCAAGTGTTCCTTGCGTTG |  |
| CAO-S | ATACTTGGATTGGCGTGCT |  |
| CAO-A | CGAAACCCTTGCTTCAGATA |  |
| CHLH-S | TCTTCACACAGACGAACCCG |  |
| CHLH-A | TCTCTCCTTCTCCACCGCAT |  |
| HEMA1-S | GCACAAAAGTGGTGGTAGTCA |  |
| HEMA1-A | CTGGTAAACACAACATCCGC |  |
| FT-S | GTAAGCAGAGTTGTTGGAGACG |  |
| FT-A | AGCCATTAGTCACCTCTCTTTG |  |
| CO-S | GGAGATAGAGTTGTTCCGCTT |  |
| CO-A | TTTGGGCGTTCTTGGGTGTG |  |
| SVP-S | CGAAACGAAGAAGAGGGCT |  |
| SVP-A | GCTGTAACTCAAGAGATGGCTG |  |
| LFY-S | GAGGTAGTGGTTTGGGGACA |  |
| LFY-A | GAAGAAGGAACTCACGGCAT |  |
| LHY-S | CAACAAGTCAACGACAATCACCAAT |  |
| LHY-A | GAAAGCCCGACGAGAAACT |  |
| SOC1-S | GAAGAGAATAGAGAATGCAACAAGC |  |
| SOC1-A | TTTGCCTTTAGGAGAGAAGATGATA |  |
| AtSEP1-S | AGGAGGATGGGAAGGTGGTGAA |  |
| AtSEP1-A | GCTTGGGTTGTCGCAGTTAT |  |
| AtSEP2-S | TAGGAGGAGGATGGGAAGGT |  |
| AtSEP2-A | AGCCAGGGATGTAGCCGTTT |  |
| AtSEP3-S | GCTCTCAGGACACAGTTTATGC |  |
| AtSEP3-A | TCCTGCTCCCATTCCATCT |  |
| AtSEP4-S | CTACCAAGGCTCGGTCTATG |  |
| AtSEP4-A | CCCAAAACGATTGAGTAAGTGC |  |
| AtActin-S | GGTAACATTGTGCTCAGTGGTGG |  |
| AtActin-A | AACGACCTTAATCTTCATGCTGC |  |
| ZjActin-S | AGCCTTCCTGCCAACGAGT |  |
| ZjActin-A | TTGCTTCTCACCCTTGATGC |  |
| ZjLHY-S | ATGGAGGCATATTCCTCCGGTGAAGATTTGG | Clone |
| ZjLHY-A | TCAAGTTGAAGCTTCCCCTTCCAAGCGCAACCTC |  |
| PpLHY-S | ATGGAAAGTTGGAAACAACTTTCAGTTTCCAACC |  |
| PpLHY-A | TCAAGTTTGAGCTTCCCCTTCCAAGCGTAACC |  |
| PbLHY-S | ATGGGACAATCAATTGACATAGATATCCCGCCTCC |  |
| PbLHY-A | TCAAGTTTGAGCTTCCCCTTCTAAGCGTAACCTC |  |
| MdLHY-S | ATGGACACAAACTCATCTGGAGAAGACCTTTTC |  |
| MdLHY-A | TCAAGTTTGAGCTTCCCCTTCTAAGCGTAACCTC |  |
| MBP-SEP3-S | AGGGAAGGATTTCAGATGGGAAGAGGTAGAGTGGAG | EMSA |
| MBP-SEP3-A | TAGAGGATCCGAATTTCATGGTAACCATCCTGGCATG |  |
| N-ZjSEP3-S | GGCGCGCCACTAGTGATGGGAAGAGGTAGAGTGGAG | BIFC |
| N-ZjSEP3-A | TACTATCGATGGATCTCATGGTAACCATCCTGGCATGT |  |
| C-AtLHY-S | GGCGCGCCACTAGTGATGGAAGAGCTTGGCAACGAAT |  |
| C-AtLHY-A | TACTATCGATGGATCTGTAGAAGCTTCTCCTTCCAAT |  |
| GFP-ZjSEP3-S | AGCTCGGGTACCCGGGATGGGAAGAGGTAGAGTGGAG | Subcellular localization |
| GFP-ZjSEP3-A | TCGACTCTAGAGGATCTGGTAACCATCCTGGCATGT |  |
| 3301AtLHY-S | TCCATGGAAGAGCTTGGCAACGA | Transgenic Arabidopsis |
| 3301AtLHY-A | TGGTNACCTCATGTAGAAGCTTCTCCTTCC |  |
|  |  |  |
