# Supplementary material for: ZjSEP3 modulates flowering time by regulating the LHY promoter
Source: BMC Plant Biol. 2021 Nov 11;21:527. doi: 10.1186/s12870-021-03305-x (PMC8582215; doi:10.1186/s12870-021-03305-x)
Supplement: Supplementary file 9 — Additional file 9: Table S3. DNA fragments used in Y1H. [file 12870_2021_3305_MOESM9_ESM.docx]

| **Table S3 DNA fragments used in Y1H** | |
| --- | --- |
| **Name** | **Sequence** |
| pC1 | CTCTCACTCTCACTCTCACGGTGGGGCTTCCCACAACAACAACGATCAAGACAAGGCCAATAATTGGCTTCTTGGATGTCTTTATTTAACCATAGGAACAGT |
| pC2 | CAAAGAAACGGAATCATCAACTGCATTGTCTTCAGGAATGGATAACGAAGCTCAATATACTACTCCTAATAAGGATAACGACTCTAAGTCGCCCGTTTAATTTAATTATAT |
| pC3 | CTTGGTGCTGTTCCAGCCTCAAATAAACTTTTCAATTAAAATTTTTCCAAAAATTAGGGGAAAAATTGTTGTGGCTGAGATTGCTTCTGGCTTCTCTTCTTCTTCTTCCAGTCTTCTTCA |
